# Supplementary material for: Untreated hypertension in Russian 35-69 year olds – a cross-sectional study
Source: PLoS One. 2020 May 29;15(5):e0233801. doi: 10.1371/journal.pone.0233801 (PMC7259637; doi:10.1371/journal.pone.0233801)
Supplement: S1 Table — (DOCX) [file pone.0233801.s001.docx]

*Table S1 Characteristics of female study participants: treated versus untreated hypertension (N=1,265). Age-standardised to 2013 European Standard Population.*

| **Characteristic** | **Level** | **Treated** |  | **Untreated** |  |
| --- | --- | --- | --- | --- | --- |
|  |  | **N** | **%** | **N** | **%** |
| Study total | Total | 991 | 100 | 274 | 100 |
| Age group | 35-49 yr | 142 | 14.3 | 78 | 28.5 |
|  | 50-59 yr | 304 | 30.7 | 97 | 35.4 |
|  | 60-69 yr | 545 | 55.0 | 99 | 36.1 |
| Education | Elementary | 75 | 5.7 | 12 | 3.7 |
|  | Lower intermediate | 182 | 14.2 | 45 | 16.1 |
|  | Higher intermediate | 454 | 45.0 | 121 | 40.7 |
|  | Graduate | 280 | 35.1 | 96 | 39.6 |
| Economic activity | Paid work | 142 | 37.4 | 81 | 43.4 |
|  | Looking after home | 131 | 10.7 | 27 | 9.9 |
|  | Unemployed | 11 | 2.7 | 3 | 1.6 |
|  | Retired | 703 | 48.4 | 160 | 43.9 |
|  | Other | 4 | 0.8 | 3 | 1.3 |
| Household income | Constrained | 247 | 22.4 | 66 | 21.9 |
|  | Intermediary | 516 | 52.6 | 129 | 47.7 |
|  | Rel. unconstrained | 214 | 24.9 | 75 | 30.4 |
| Single | No | 520 | 57.5 | 161 | 59.8 |
|  | Yes | 471 | 42.5 | 113 | 40.2 |
| Smoking | No | 857 | 80.2 | 222 | 78.9 |
|  | Yes | 133 | 19.8 | 52 | 21.1 |
| Alcohol use disorder | Non-drinker past year | 868 | 81.6 | 218 | 73.6 |
|  | Low (AUDIT<8) | 109 | 16.5 | 41 | 20.1 |
|  | High (AUDIT 8+) | 12 | 1.8 | 15 | 6.3 |
| Physical activity | Inactive | 49 | 6.4 | 13 | 5.5 |
|  | Moderately inactive | 88 | 13.5 | 31 | 13.6 |
|  | Moderately active | 639 | 53.9 | 166 | 57.1 |
|  | Active | 197 | 26.3 | 59 | 23.8 |
| Self-rated general health | Poor/fair/good | 767 | 70.1 | 160 | 57.2 |
|  | Very good/excellent | 223 | 29.9 | 114 | 42.8 |
| Body Mass Index | Under/Normal (<25) | 133 | 18.1 | 56 | 21.3 |
|  | Overweight (25-29) | 308 | 28.9 | 115 | 43.6 |
|  | Obese (30-34) | 321 | 30.4 | 71 | 24.2 |
|  | Very obese (35+) | 227 | 22.7 | 31 | 10.9 |
| Hypertension aware | No | 113 | 15.0 | 161 | 60.0 |
|  | Yes | 878 | 85.0 | 113 | 40.0 |
| Hypertension knowledge | Thinks symptoms always present | 422 | 43.0 | 103 | 38.3 |
|  | Thinks symptoms sometimes present | 449 | 48.8 | 125 | 48.0 |
|  | Think symptoms never present | 115 | 8.2 | 42 | 13.7 |
| Diabetic | No | 786 | 85.2 | 254 | 94.2 |
|  | Yes | 205 | 14.8 | 20 | 5.8 |
| CKD | No | 908 | 95.3 | 262 | 96.9 |
|  | Yes | 83 | 4.7 | 12 | 3.1 |
| CVD history | No | 595 | 70.2 | 239 | 89.4 |
|  | Yes | 396 | 29.8 | 35 | 10.6 |
| Seen primary care doctor past year | No | 159 | 17.8 | 109 | 38.6 |
|  | Yes | 832 | 82.2 | 165 | 61.4 |
| General health check attendance | No | 366 | 43.1 | 135 | 50.1 |
|  | Yes | 625 | 56.9 | 139 | 49.9 |
