# Supplementary material for: Untreated hypertension in Russian 35-69 year olds – a cross-sectional study
Source: PLoS One. 2020 May 29;15(5):e0233801. doi: 10.1371/journal.pone.0233801 (PMC7259637; doi:10.1371/journal.pone.0233801)
Supplement: S2 Table — (DOCX) [file pone.0233801.s002.docx]

*Table S2 Characteristics of male study participants: treated versus untreated hypertension (N=1,088). Age-standardised to 2013 European Standard Population.*

| **Characteristic** | **Level** | **Treated** |  | **Untreated** |  |
| --- | --- | --- | --- | --- | --- |
|  |  | **N** | **%** | **N** | **%** |
| Study total | Total | 613 | 100 | 475 | 100 |
| Age group | 35-49 yr | 95 | 15.5 | 161 | 33.9 |
|  | 50-59 yr | 185 | 30.2 | 151 | 31.8 |
|  | 60-69 yr | 333 | 54.3 | 163 | 34.3 |
| Education | Elementary | 54 | 7.3 | 53 | 10.6 |
|  | Lower intermediate | 139 | 22.6 | 105 | 21.9 |
|  | Higher intermediate | 205 | 34.9 | 165 | 35.0 |
|  | Graduate | 215 | 35.2 | 152 | 32.5 |
| Economic activity | Paid work | 128 | 42.5 | 198 | 50.2 |
|  | Looking after home | 23 | 2.6 | 13 | 2.4 |
|  | Unemployed | 13 | 2.9 | 17 | 3.4 |
|  | Retired | 435 | 46.7 | 232 | 40.0 |
|  | Other | 14 | 5.2 | 15 | 4.0 |
| Household income | Constrained | 121 | 20.5 | 82 | 16.9 |
|  | Intermediary | 296 | 48.6 | 222 | 48.0 |
|  | Rel. unconstrained | 189 | 30.9 | 160 | 35.2 |
| Single | No | 535 | 85.4 | 391 | 82.0 |
|  | Yes | 78 | 14.6 | 84 | 18.0 |
| Smoking | No | 415 | 60.7 | 276 | 57.5 |
|  | Yes | 197 | 39.3 | 196 | 42.5 |
| Alcohol use disorder | Non-drinker past year | 286 | 41.7 | 173 | 32.8 |
|  | Low (AUDIT<8) | 184 | 32.6 | 157 | 34.7 |
|  | High (AUDIT 8+) | 141 | 25.7 | 140 | 32.5 |
| Physical activity | Inactive | 22 | 4.7 | 26 | 5.7 |
|  | Moderately inactive | 82 | 14.1 | 49 | 9.7 |
|  | Moderately active | 366 | 51.4 | 230 | 48.5 |
|  | Active | 132 | 29.8 | 161 | 36.2 |
| Self-rated general health | Poor/fair/good | 399 | 60.0 | 203 | 41.7 |
|  | Very good/excellent | 213 | 40.0 | 270 | 58.3 |
| Body Mass Index | Under/Normal (<25) | 100 | 15.3 | 137 | 28.1 |
|  | Overweight (25-29) | 273 | 43.1 | 231 | 48.8 |
|  | Obese (30-34) | 173 | 28.2 | 84 | 18.1 |
|  | Very obese (35+) | 65 | 13.4 | 22 | 5.0 |
| Hypertension aware | No | 84 | 14.7 | 292 | 62.5 |
|  | Yes | 529 | 85.3 | 183 | 37.5 |
| Hypertension knowledge | Thinks symptoms always present | 213 | 36.2 | 179 | 42.0 |
|  | Thinks symptoms sometimes present | 283 | 50.2 | 181 | 41.0 |
|  | Think symptoms never present | 111 | 13.6 | 87 | 17.0 |
| Diabetic | No | 499 | 85.1 | 451 | 95.5 |
|  | Yes | 114 | 14.9 | 24 | 4.5 |
| CKD | No | 575 | 95.1 | 466 | 98.5 |
|  | Yes | 38 | 4.9 | 9 | 1.5 |
| CVD history | No | 337 | 64.3 | 410 | 88.0 |
|  | Yes | 276 | 35.7 | 65 | 12.0 |
| Seen primary care doctor past year | No | 150 | 24.6 | 228 | 48.1 |
|  | Yes | 463 | 75.4 | 247 | 51.9 |
| General health check attendance | No | 362 | 66.6 | 334 | 70.7 |
|  | Yes | 251 | 33.4 | 141 | 29.3 |
