# Supplementary material for: Untreated hypertension in Russian 35-69 year olds – a cross-sectional study
Source: PLoS One. 2020 May 29;15(5):e0233801. doi: 10.1371/journal.pone.0233801 (PMC7259637; doi:10.1371/journal.pone.0233801)
Supplement: S3 Table — (DOCX) [file pone.0233801.s003.docx]

*Table S3 Prevalence (%) of untreated hypertension among hypertensive 35-69 year olds (age- and gender-standardised to European standard population) with 95% confidence intervals.*

| Group | Untreated hypertension% | Untreated hypertension – Aware% | Untreated hypertension – Unaware% |
| --- | --- | --- | --- |
|  |  |  |  |
| Male | 51.1 (47.8-54.5) | 18.6 (16.0-21.5) | 32.5 (29.3-36.0) |
| Female | 28.8 (25.4-32.5) | 11.3 (8.9-14.2) | 17.6 (14.6-20.9) |
| Total | 40.0 (37.5-42.5) | 14.8 (13.0-16.8) | 25.2 (22.9-27.6) |
| Total (sensitivity)* | 47.3 (44.8-49.9) | 17.5 (15.5-19.7) | 29.8 (27.4-32.4) |

*) Sensitivity analysis based on self-reported antihypertensive use where the main analyses were based on ATC coding of participant medication.
