# Supplementary material for: Untreated hypertension in Russian 35-69 year olds – a cross-sectional study
Source: PLoS One. 2020 May 29;15(5):e0233801. doi: 10.1371/journal.pone.0233801 (PMC7259637; doi:10.1371/journal.pone.0233801)
Supplement: S4 Table — (DOCX) [file pone.0233801.s004.docx]

*Table S4 Characteristics of study participants with any hypertension status incl. normotensive (N=4,102): without repeated measurement versus with repeated measurement.*

| **Characteristic** | **Level** | **Without repeated** |  | **With repeated** |  |
| --- | --- | --- | --- | --- | --- |
|  |  | **N** | **%** | **N** | **%** |
| Study total | Total | 3,770 | 100 | 332 | 100 |
| Age group | 35-49 yr | 1,322 | 35.1 | 112 | 33.7 |
|  | 50-59 yr | 1,136 | 30.1 | 96 | 28.9 |
|  | 60-69 yr | 1,312 | 34.8 | 124 | 37.4 |
|  | Mean age (SD) | 54.4 | (9.7) | 54.8 | (9.5) |
| Gender | Male | 1,561 | 41.4 | 154 | 46.4 |
|  | Female | 2,209 | 58.6 | 178 | 53.6 |
| Education | Elementary | 256 | 6.8 | 29 | 8.7 |
|  | Lower intermediate | 684 | 18.1 | 47 | 14.2 |
|  | Higher intermediate | 1,415 | 37.5 | 122 | 36.8 |
|  | Graduate | 1,415 | 37.5 | 134 | 40.4 |
| Economic activity | Paid work | 1,328 | 35.2 | 112 | 33.8 |
|  | Looking after home | 285 | 7.6 | 21 | 6.3 |
|  | Unemployed | 94 | 2.5 | 11 | 3.3 |
|  | Retired | 1,972 | 52.3 | 182 | 55.0 |
|  | Other | 89 | 2.4 | 5 | 1.5 |
| Household income | Constrained/low | 719 | 19.4 | 61 | 18.5 |
|  | Intermediary | 1,833 | 49.5 | 176 | 53.3 |
|  | Rel. unconstrained/high | 1,150 | 31.1 | 93 | 28.2 |
| Single | No | 2,612 | 69.3 | 232 | 69.9 |
|  | Yes | 1,158 | 30.7 | 100 | 30.1 |
| Smoking | No | 2,816 | 74.8 | 239 | 72.4 |
|  | Yes | 947 | 25.2 | 91 | 27.6 |
| Alcohol use disorder | Non-drinker past year | 2,536 | 67.5 | 205 | 62.3 |
|  | Low (AUDIT<8) | 798 | 21.2 | 85 | 25.8 |
|  | High (AUDIT 8+) | 424 | 11.3 | 39 | 11.9 |
| Physical activity | Inactive | 214 | 5.8 | 20 | 6.1 |
|  | Moderately inactive | 392 | 10.6 | 34 | 10.4 |
|  | Moderately active | 2,118 | 57.1 | 200 | 61.0 |
|  | Active | 988 | 26.6 | 74 | 22.6 |
| Self-rated general health | Poor/fair/good | 2,114 | 56.2 | 189 | 57.3 |
|  | Very good/excellent | 1,649 | 43.8 | 141 | 42.7 |
| Body Mass Index | Under/Normal (<25) | 1,150 | 30.6 | 98 | 29.6 |
|  | Overweight (25-29) | 1,390 | 37.0 | 132 | 39.9 |
|  | Obese (30-34) | 816 | 21.7 | 68 | 20.5 |
|  | Very obese (35+) | 406 | 10.8 | 33 | 10.0 |
| Hypertension aware | No | 1,937 | 51.4 | 155 | 46.7 |
|  | Yes | 1,833 | 48.6 | 177 | 53.3 |
| Diabetic | No | 3,385 | 89.8 | 288 | 86.8 |
|  | Yes | 385 | 10.2 | 44 | 13.3 |
| CKD | No | 3,616 | 95.9 | 313 | 94.3 |
|  | Yes | 154 | 4.1 | 19 | 5.7 |
| CVD history | No | 2,828 | 75.0 | 243 | 73.2 |
|  | Yes | 942 | 25.0 | 89 | 26.8 |
| Seen GP/Polyclinic doctor past year | No | 1,137 | 30.2 | 76 | 22.9 |
|  | Yes | 2,633 | 69.8 | 256 | 77.1 |
| General health check attendance | No | 2,011 | 53.3 | 171 | 51.5 |
|  | Yes | 1,759 | 46.7 | 161 | 48.5 |
