# Supplementary material for: Untreated hypertension in Russian 35-69 year olds – a cross-sectional study
Source: PLoS One. 2020 May 29;15(5):e0233801. doi: 10.1371/journal.pone.0233801 (PMC7259637; doi:10.1371/journal.pone.0233801)
Supplement: Attachment — Submitted filename: Response to reviewers.docx [file pone.0233801.s005.docx]

PLOS ONE

Re: Untreated hypertension in Russian 35-69 year olds – a cross-sectional study (PONE-D-19-34971)

Dear Dr Bart Ferket,

Please find our response to the reviewers’ comments below. We found the comments constructive and feel that the process has strengthened the paper.

Many regards and on behalf of the author team,

Jakob Petersen

London, 03 March 2020

Attachments: Response to Reviewers, Revised Manuscript with Track Changes, Manuscript

Editor’s comments

**I agree with both reviewers your manuscript can benefit from subheadings and better structuring of the Methods section.**

Response:

*We agree that the structure and content suggested by the reviewers will make the Methods section much clearer. We have now restructured and expanded it accordingly.*

**Also provide more details on study population selection and expand the discussion section on how to make inferences about generalizability of odds ratios given the difference in participation rates between the two urban populations. Are these still valid or would the estimation of odds ratios be affected due to the selection process?**

Response:

*We agree that the low response rate, especially in one of the two cities, could lead to sampling bias. We currently discuss this in the Limitations sub-section, where we refer to analyses carried out by Cook et al. (2018) in our earlier paper on the study methodology. Cook et al. compared age, sex, and higher education attainment of respondents with the Russian 2010 Census population of each city. The results showed that the sample attending for the health check was almost the same as that in the Census, with a ratio of 0.99 (95% CI 0.93-1.06) for Arkhangelsk and 1.26 (1.17-1.34) for Novosibirsk.*

*We also note that our prevalence estimates of untreated hypertension were very close to the concurrent ESSE-RF Study of 25-64 year olds in four other Russian regions (Balanova et al. 2019).*

*We feel that the relatively low response rate needs to be acknowledged when interpreting the results. Based on the comparison with the Russian Census population we have no good reason to conclude that the respondents differed markedly in terms of age, sex, and higher education attainment.*

**Reviewer 2 makes an important point about including psychosocial CVD risk factors such as depression, anxiety and stress. These variables should be included in the multivariable analysis when available.**

Response:

*We agree and now include a reference in support of this suggestion and have included depression status (PHQ-9 instrument) in the analyses. Given that the constructs for depression, anxiety, and stress are related, we suggest that only the most severe of the three, i.e. depression, is included.*

**Please also provide results from a logistic regression model including all variables (not only adjusted for age and CVD, but adjusting with all other variables) and test for interaction by gender (within one comprehensive model, not separated by gender).**

Response:

*We present the joint model for both genders below (age- and sex-adjusted and fully adjusted odds ratios). No significant interactions between age group and gender were found. This was tested with a likelihood ratio test in the full model without an interaction term versus the full model with an interaction term.*

*It is well known that men and women differ in health behaviours, for many reasons including gender norms and expectations and targeted marketing of unhealthy products, and we suggest to keep the gender-specific regression tables.*

Table	Logistic regression models of untreated versus treated hypertension. Age- and fully adjusted odds ratios (AOR) with 95% confidence intervals.

| **Characteristic** | **Level** | **AOR age/sex** | **P-value** | **95% CI** | **AOR all** | **P-value** | **95% CI** |
| --- | --- | --- | --- | --- | --- | --- | --- |
| Gender | Female | Ref |  |  | Ref |  |  |
|  | Male | **2.74** | <.001 | 2.28-3.29 | **1.67** | <.001 | 1.28-2.19 |
| CVD history | No | Ref |  |  | Ref |  |  |
|  | Yes | **0.24** | <.001 | 0.19-0.31 | **0.31** | <.001 | 0.24-0.40 |
| Age group | 35-49 yr | Ref |  |  | Ref |  |  |
|  | 50-59 yr | **0.53** | <.001 | 0.41-0.67 | **0.71** | .034 | 0.51-0.97 |
|  | 60-69 yr | **0.31** | <.001 | 0.24-0.39 | **0.63** | .017 | 0.43-0.92 |
| Education | Elementary | Ref |  |  | Ref |  |  |
|  | Lower intermediate | 0.84 | .368 | 0.58-1.23 | 0.85 | .450 | 0.56-1.30 |
|  | Higher intermediate | 0.83 | .290 | 0.58-1.18 | 0.80 | .265 | 0.54-1.19 |
|  | Graduate | 0.84 | .336 | 0.59-1.20 | 0.71 | .106 | 0.47-1.07 |
| Economic activity | Retired | Ref |  |  | Ref |  |  |
|  | Paid work | **1.71** | <.001 | 1.28-2.29 | 1.39 | .051 | 1.00-1.94 |
|  | Looking after home | 0.96 | .833 | 0.66-1.40 | 0.89 | .599 | 0.58-1.37 |
|  | Unemployed | 1.41 | .286 | 0.75-2.67 | 1.76 | .132 | 0.84-3.67 |
|  | Other | 1.38 | .369 | 0.68-2.81 | 0.92 | .848 | 0.41-2.09 |
| Household income | Constrained | Ref |  |  | Ref |  |  |
|  | Intermediary | 0.97 | .801 | 0.76-1.23 | 0.83 | .181 | 0.63-1.09 |
|  | Rel. unconstrained | 1.11 | .437 | 0.85-1.45 | 0.87 | .393 | 0.63-1.20 |
| Single | No | Ref |  |  | Ref |  |  |
|  | Yes | 1.04 | .734 | 0.84-1.29 | 1.02 | .876 | 0.80-1.30 |
| Smoking | No | Ref |  |  | Ref |  |  |
|  | Yes | **1.32** | .009 | 1.07-1.63 | 1.14 | .305 | 0.89-1.46 |
| Alcohol use disorder | Non-drinker past year | Ref |  |  | Ref |  |  |
|  | Low (AUDIT<8) | 1.21 | .108 | 0.96-1.54 | 0.93 | .619 | 0.68-1.26 |
|  | High (AUDIT 8+) | **1.49** | .006 | 1.12-1.98 | 1.00 | .996 | 0.68-1.47 |
| Physical activity | Inactive | Ref |  |  | Ref |  |  |
|  | Moderately inactive | 0.87 | .569 | 0.53-1.42 | 1.13 | .668 | 0.64-1.99 |
|  | Moderately active | 0.91 | .655 | 0.59-1.40 | 1.09 | .734 | 0.66-1.79 |
|  | Active | 1.10 | .672 | 0.70-1.73 | 1.08 | .775 | 0.64-1.81 |
| Self-rated general health | Poor/fair/good | Ref |  |  | Ref |  |  |
|  | Very good/excellent | **2.19** | <.001 | 1.81-2.65 | **1.48** | .001 | 1.18-1.84 |
| Body Mass Index | Under/Normal (<25) | Ref |  |  | Ref |  |  |
|  | Overweight (25-29) | **0.73** | .012 | 0.57-0.93 | 0.77 | .054 | 0.59-1.00 |
|  | Obese (30-34) | **0.43** | <.001 | 0.33-0.56 | **0.48** | <.001 | 0.35-0.65 |
|  | Very obese (35+) | **0.26** | <.001 | 0.18-0.38 | **0.35** | <.001 | 0.23-0.52 |
| Diabetic | No | Ref |  |  | Ref |  |  |
|  | Yes | **0.30** | <.001 | 0.22-0.42 | **0.47** | <.001 | 0.32-0.68 |
| CKD | No | Ref |  |  | Ref |  |  |
|  | Yes | **0.50** | .005 | 0.31-0.81 | 0.66 | .131 | 0.39-1.13 |
| Depression (PHQ-9) | No | Ref |  |  | Ref |  |  |
|  | Yes | **0.68** | <.001 | 0.56-0.83 | 0.88 | .288 | 0.69-1.11 |
| Seen primary care doctor past year | No | Ref |  |  | Ref |  |  |
|  | Yes | **0.32** | <.001 | 0.26-0.39 | **0.38** | <.001 | 0.31-0.48 |

Reviewers' comments:

Reviewer #1:

**The aim of the study is not clearly stated. I just assume it was (1) to determine the prevalence of untreated hypertension in population samples of Arkhangelsk and Novosibirsk and (2) to identify factors to be associated with untreated hypertension in the two population samples.**

Response:

*The reviewer is correct and we have amended the Introduction accordingly.*

**Section Materials and methods should be clearly structured having the following subsections: Population (this should also include how the individuals were selected), Screening examination (should be briefly described, including blood pressure measurement), Definition of risk factors (incl. hypertension).**

Response:

*We agree that the suggested structure will make the Methods section much clearer. We have now restructured and expanded the section accordingly.*

**The definition of hypertension used on lines 117 and 118 does not seem to include values equal to 140/90 mmHg, which is not in agreement with the quoted European guidelines on hypertension.**

Response:

*We have now phrased this with words, i.e. ‘at or above 140’ instead of ‘140+’. In this way it should be clearer that the guideline definition was indeed adhered to.*

Reviewer #2:

The submitted manuscript assesses the rate of untreated hypertension based on the «Know Your Heart» study and is carried out in two Russian cities, namely Novosibirsk and Arkhangelsk. The aim was also to provide further insights into the country-specific factors contributing to the fact that far too many hypertensive men and women currently remain untreated. However, there are a number of points, which need clarifications and revision.

**1) The Materials and Methods section is not enough conclusive. It is difficult to understand the methodology of the study. Although authors provided a reference to their comprehensive paper on the «Know Your Heart» study design [Cook S, Malyutina S, Kudryavtsev AV, et al. Know Your Heart: Rationale, design and conduct of a cross-sectional study of cardiovascular structure, function and risk factors in 4500 men and women aged 35-69 years from two Russian cities, 2015-18. Wellcome Open Res. 2018;3:67.], it is not enough. I recommend to give in the current paper at least basic explanations on how the study population was selected.**

Response:

*Following the restructuring of the Methods section as suggested by Reviewer 1, we now also include a brief overview of the sampling and study components under the heading of ‘Population’.*

**2) The description of statistical methods is very scarce; they are presented literally in a couple of sentences and are not allocated in a separate subsection.**

Response:

*In the new structure of the Methods section, we now bring together the descriptions of the statistical analysis in a new sub-section.*

**3) The robustness of results seems doubtful due to a low response rate. Moreover, the response rate in Novosibirsk, which is a developed city with a population of more than 1.5 million people, was more than two times lower than in Arkhangelsk (22% vs 51%, respectively). This issue must be addressed in some way.**

Response:

*Please see response to Editor’s comments above.*

**4) Within the scope of the studied problems, the psychosocial CVD risk factors such as depression, anxiety and stress are shown to be of great importance for the Russian population. These factors serve as barriers for traditional risk factors modification and CVD treatment, including hypertension treatment, and they are also known to independently affect long-term health outcomes in CVD patients. According to the design paper, the “Know Your Heart” study encompassed assessment of anxiety and depression (using validated questionnaires), as well as assessment of self-reported social support. It is not clear, why these data were not used in the multivariate logistic regression models of untreated vs treated hypertension in this paper. The inclusion of these data into the models may provide more accurate and comprehensive information on factors, associated with lack of antihypertensive treatment, specifically relevant for the Russian population.**

Response:

*We have included depression status (PHQ-9 instrument) in the analyses. Given that the constructs for depression, anxiety, and stress are related, we suggest that only the most severe of the three, i.e. depression, to be included. The results show that depression is borderline protective for untreated hypertension. This is consistent with the observation that hypertension is asymptomatic and less likely to be detected in individuals who are not in contact with the health system. Having other conditions, in this case, depression, may increase the probability of health system contact and treatment. While depression has been shown to affect individuals functionally in many ways, it cannot be concluded that it is a driver of untreated hypertension in the population at large.*

**5) I would recommend avoiding inappropriate slang expressions like antihypertensives instead of antihypertensive drugs or medicines or treatment.**

Response:

*We now use the term, ‘antihypertensive medications’, throughout for clarity.*
